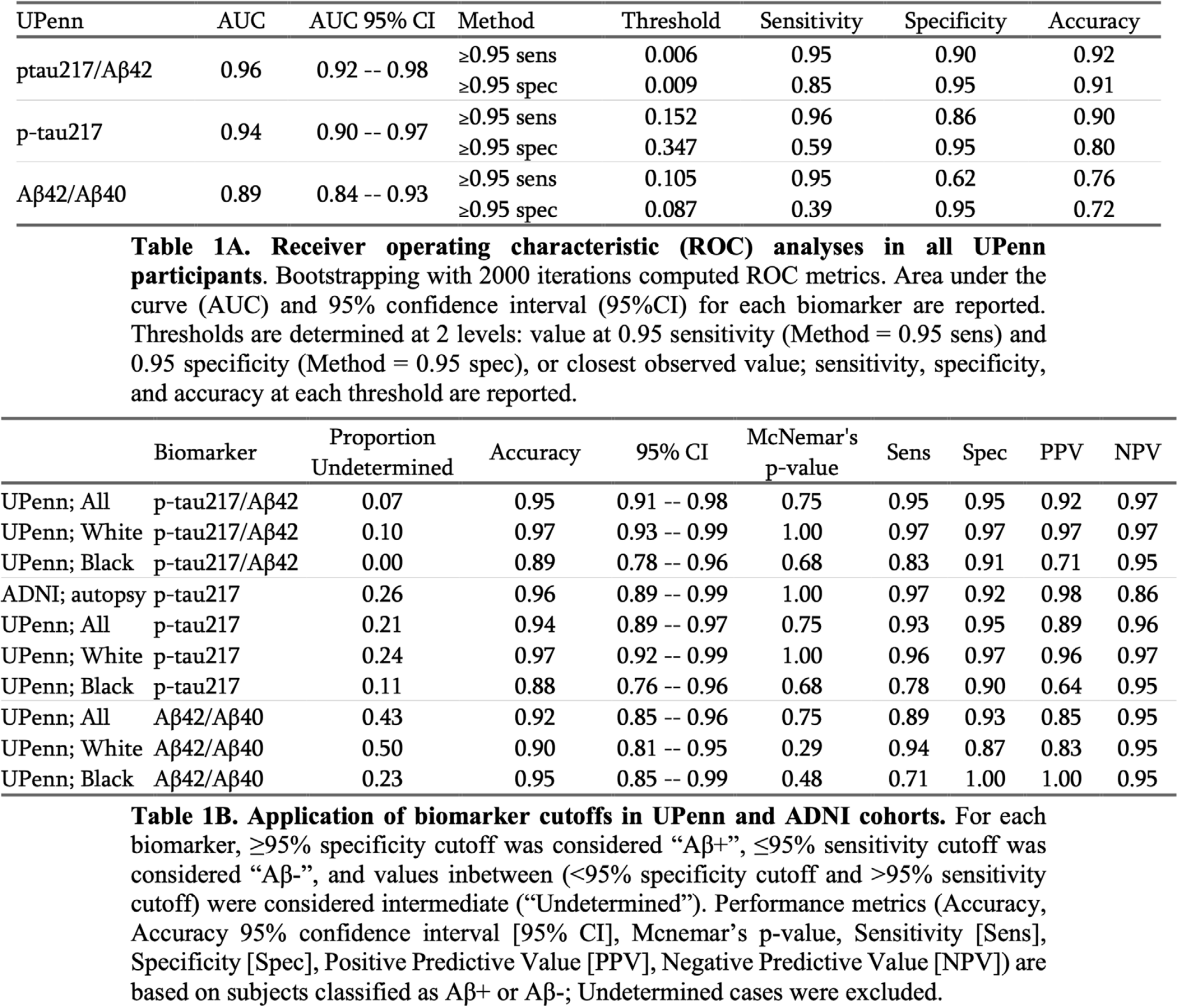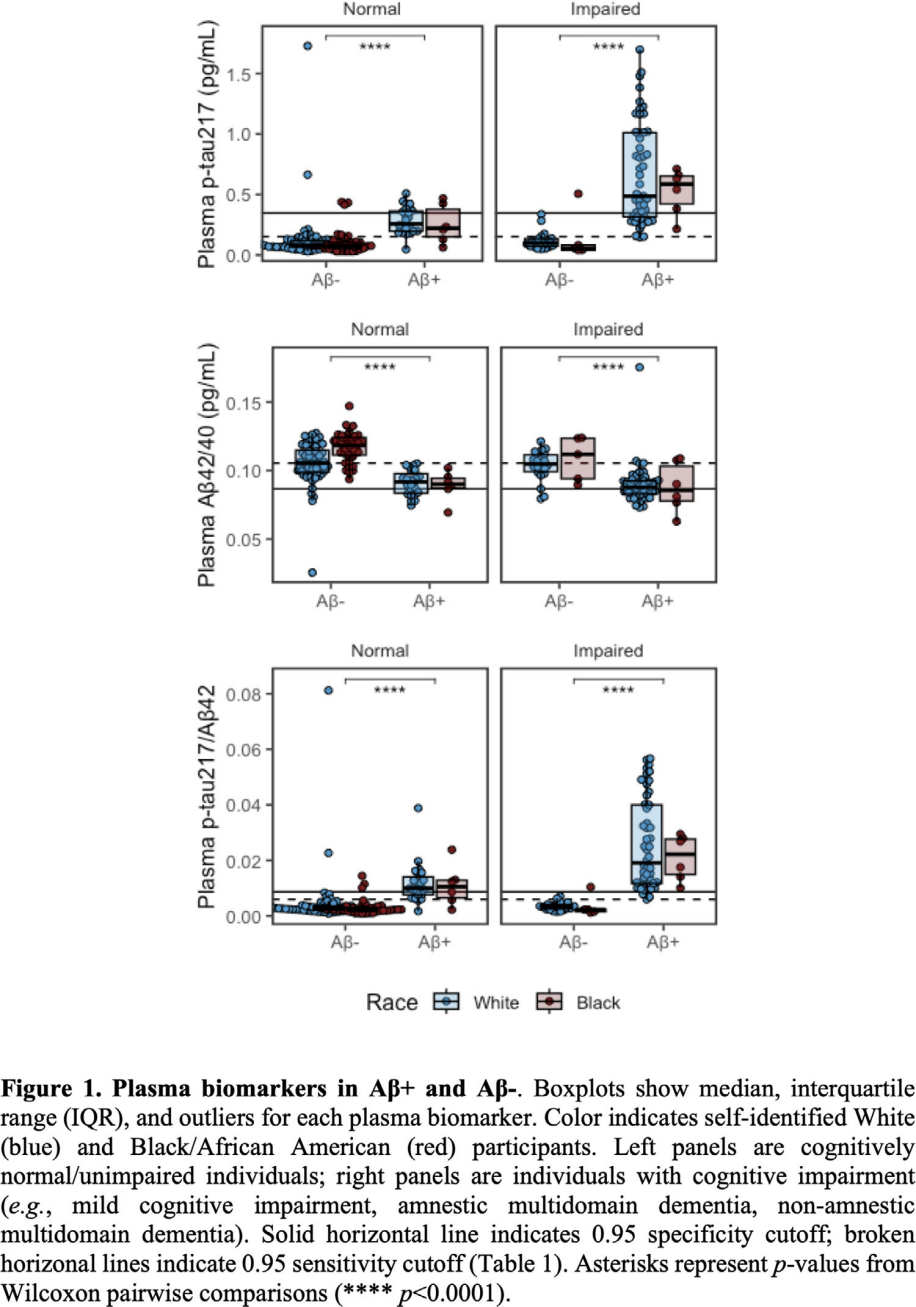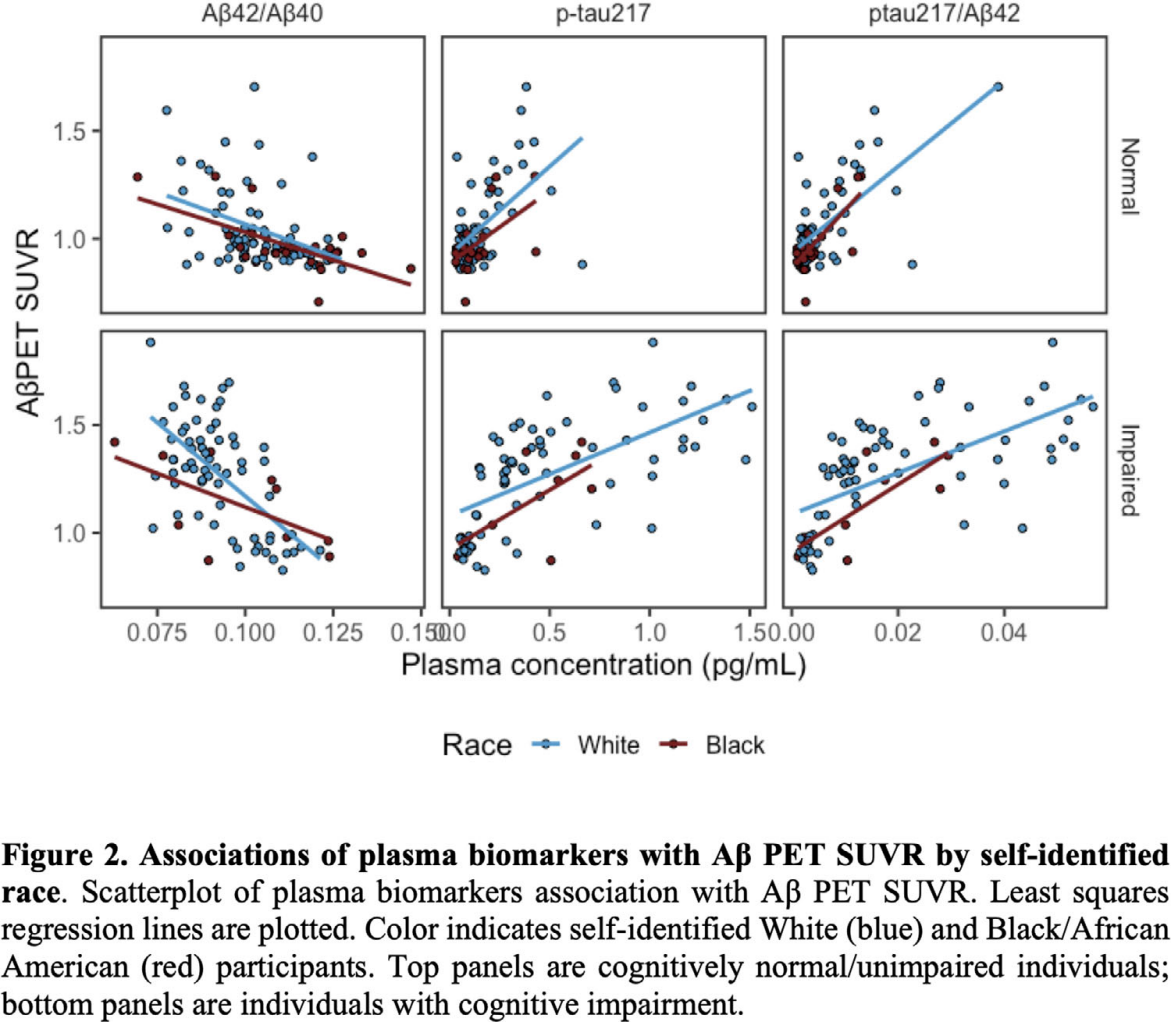# Comparison of plasma p‐tau_217_ and Aβ_42_/Aβ_40_ biomarkers by race to detect Alzheimer’s disease

**DOI:** 10.1002/alz.094605

**Published:** 2025-01-09

**Authors:** Katheryn A Q Cousins, Magdalena Korecka, Wan Yang, Amberley Vulaj, Christopher Brown, Thomas F. Tropea, Alice Chen‐Plotkin, Eddie B Lee, David J Irwin, David A Wolk, Leslie M. Shaw

**Affiliations:** ^1^ Department of Neurology, University of Pennsylvania, Philadelphia, PA USA; ^2^ Perelman School of Medicine, University of Pennsylvania, Department of Pathology and Laboratory Medicine, Philadelphia, PA USA; ^3^ University of Pennsylvania, Philadelphia, PA USA; ^4^ Perelman School of Medicine, University of Pennsylvania, Philadelphia, PA USA; ^5^ Center for Neurodegenerative Disease Research, Perelman School of Medicine, University of Pennsylvania, Philadelphia, PA USA; ^6^ Penn Frontotemporal Degeneration Center, Department of Neurology, Perelman School of Medicine, University of Pennsylvania, Philadelphia, PA USA; ^7^ Penn Alzheimer’s Disease Research Center, University of Pennsylvania, Philadelphia, PA USA; ^8^ Dept of Pathology & Laboratory Medicine, University of Pennsylvania, Perelman School of Medicine, Philadelphia, PA USA

## Abstract

**Background:**

To guide blood biomarker implementation to detect Alzheimer’s disease (AD), evaluations in clinically and racially diverse samples are needed. Here, we compare plasma biomarkers phosphorylated tau 217 (p‐tau_217_), β‐amyloid (Aβ) 1‐42/1‐40 (Aβ_42_/Aβ_40_) ratio, and p‐tau_217_/Aβ_42_ ratio in a University of Pennsylvania (UPenn) sample that includes self‐identified White and Black/African American individuals with and without cognitive impairment, and independently validate findings in the first 100 AD Neuroimaging Initiative (ADNI) autopsy participants.

**Methods:**

UPenn inclusion criteria were plasma p‐tau_217_, Aβ_42_, and Aβ_40_ (n = 213) assayed using the automated Fujirebio platform, self‐reported race (156 White [including 1 Hispanic White, 2 multiracial] and 57 Black), and ^18^F‐florbetaben PET scan to determine Aβ positivity by visual read (87 Aβ+, 126 Aβ‐). Participants were cognitively Normal (n = 128) or Impaired (n = 85) according to National Alzheimer’s Coordinating Center (NACC) Uniform Dataset 3.0 criteria. Linear models tested if plasma biomarkers (dependent variable, log‐transformed) differed by race and/or Aβ PET status (±). To confirm observed racial differences, linear models tested the interaction of race by global Aβ PET standardized uptake value ratio (SUVR). Models covaried for age at plasma, sex, 2021 national area deprivation index (ADI), and APOE ε4. Receiver operating characteristic (ROC) with bootstrapping (2000 iterations) compared biomarker performance (area under the curve [AUC]) and calculated 2 thresholds at 0.95 sensitivity and 0.95 specificity. Plasma p‐tau_217_ cutpoints were validated in ADNI autopsy sample (n = 100); high/intermediate AD neuropathologic change considered AD+.

**Results:**

P‐tau_217_/Aβ_42_ had the highest performance to discriminate Aβ+ from Aβ‐ (Table 1A,1B; all AUC≥0.90), and p‐tau_217_ showed excellent accuracy in ADNI sample (0.96; Table 1B). Linear models showed that Black individuals had significantly higher Aβ_42_ (β = 0.098, *p* = 0.0014); no difference was observed for p‐tau_217_ or p‐tau_217_/Aβ_42_ (both *p*≥0.43) (Figure 1). A Race X Aβ PET SUVR interaction confirmed this difference for plasma Aβ_42_ (β = ‐0.44, *p* = 0.00051; Figure 2).

**Conclusion:**

Findings indicate plasma p‐tau_217_ levels do not differ by race, while plasma Aβ_42_/Aβ_40_ was higher in Black individuals even after accounting for differences in Aβ PET SUVR and ADI. Future work will test biomarkers in a larger sample, and examine the influence of comorbidities on differences in plasma levels and accuracy.